# Sublethal Antibiotic Exposure Induces Microevolution of Quinolone Resistance in Pathogenic *Vibrio parahaemolyticus*

**DOI:** 10.3390/ijms27031416

**Published:** 2026-01-30

**Authors:** Qian Wu, Han Yang, Tianming Xu, Pradeep K. Malakar, Huan Li, Yong Zhao

**Affiliations:** 1 College of Food Science and Technology, Shanghai Ocean University, Shanghai 201306, China; d220300083@st.shou.edu.cn (Q.W.); h-yang@shou.edu.cn (H.Y.); d230300096@st.shou.edu.cn (T.X.); pkmalakar@shou.edu.cn (P.K.M.); 2 International Research Center for Food and Health, Shanghai Ocean University, Shanghai 201306, China; 3 Laboratory of Quality & Safety Risk Assessment for Aquatic Products on Storage and Preservation (Shanghai), Ministry of Agriculture and Rural Affairs, Shanghai 201306, China; 4 Shanghai Engineering Research Center of Aquatic-Product Processing & Preservation, Shanghai 201306, China

**Keywords:** quinolone resistance, sublethal concentration, *Vibrio parahaemolyticus*, microevolution

## Abstract

The microevolutionary pathways and molecular mechanisms by which the important pathogen *Vibrio parahaemolyticus* acquires resistance in the aquatic environment under continuous selective pressure from quinolone antibiotic residues are still unknown. Here, the study successfully simulated the long-term pressure of antibiotic residues in aquaculture by susceptible *V. parahaemolyticus* (VPD14) which was isolated from seafood, to a 30-day in vitro induction with sublethal concentrations of levofloxacin, which yielded the mutants (VPD14M). A phenotypic analysis revealed that VPD14M exhibited resistance to ampicillin, levofloxacin and ciprofloxacin, compared to VPD14. These changes were accompanied by adaptations, including a decreased growth rate and an enhanced biofilm formation capacity. Whole-Genome Sequencing identified that the acquired resistance was primarily attributable to key point mutations in three Quinolone Resistance-Determining Regions (QRDRs). Specifically, a G → T substitution at nucleotide position 248 in the *gyrA* gene, leading to a serine-to-isoleucine substitution at the 83rd amino acid position (Ser83Ile) of the DNA gyrase subunit A; a C → T substitution at position 254 in the *parC* gene, resulting in a serine-to-phenylalanine substitution at position 85 (Ser85Phe) of the topoisomerase IV subunit A; and a C → T substitution at position 2242 in the *gyrB* gene, causing a proline-to-serine substitution at position 748 (Pro748Ser) of the DNA gyrase subunit B. Collectively, the study demonstrated that sublethal antibiotic levels rapidly drive quinolone resistance in *V. parahaemolyticus*, and the specific mutations identified offer critical support for resistance monitoring and seafood safety alerts.

## 1. Introduction

The persistent presence of antibiotic residues in aquaculture environments, particularly stemming from the widespread use of quinolones, constitutes a severe global problem [1,2,3]. Notably, antibiotic residues from aquaculture can enter the environment in the form of either parent compounds or active metabolites [4,5]. These residues continuously accumulate in both the water column and sediments, exerting long-term environmental selection pressure [6]. This process not only results in the direct contamination of aquatic ecosystems but has also emerged as a key driver for the evolution of antimicrobial resistance (AMR) in aquatic microorganisms [6,7].

In turn, the emergence and dissemination of resistance further amplify the public health risks of the aforementioned environmental issues [8]. AMR has been listed by the World Health Organization (WHO) as one of the top ten global health threats. It is estimated by the U.S. Centers for Disease Control and Prevention (CDC) that antibiotic-resistant bacteria infections cause over 23,000 deaths annually in the United States. Similarly, data from the European Union similarly indicated that approximately 33,000 people die from this cause each year [9]. The emergence of resistant pathogens within the aquaculture value chain may lead to untreatable diseases in cultured animals, resulting in significant economic losses [10,11]. More critically, these pathogens can be transmitted to humans via the food chain or environmental contact, triggering infections that are difficult to cure with conventional antibiotics and thus posing a direct threat to food safety and human health [12].

This study focuses on *V. parahaemolyticus* due to its critical role as a pathogen responsible for substantial economic losses in aquaculture and as a leading cause of acute gastroenteritis worldwide [13,14,15]. With the growing severity of antibiotic resistance, quinolone resistance in this typical aquatic-origin pathogen has drawn considerable attention [16,17,18]. However, these studies lack a comprehensive understanding of the microevolutionary pathways and key molecular mechanisms by which this pathogen acquires stable resistance under stress from environmentally relevant sublethal concentrations of quinolones. In this context, Levofloxacin (LEV) was selected as the model quinolone for this study based on three key considerations: its pronounced environmental stability and persistence in aquaculture systems; its prevalent use in both veterinary and human medicine, aligning with a ‘One Health’ perspective [19]; and its mechanism of action as a dual-targeting agent against both DNA gyrase and topoisomerase IV, which enables the monitoring of concurrent evolutionary changes in multiple Quinolone Resistance-Determining Regions (QRDRs) [20].

Recently, relevant studies have been made in understanding the microevolution of bacterial antibiotic resistance when exposed to sublethal antibiotic pressure [8,21,22]. Studies using model organisms, such as *Escherichia coli*, have revealed the evolutionary pathways of adaptation following exposure to sublethal (or environmentally relevant) concentrations of quinolones, which often involve stepwise mutations [23,24]. While these studies are pioneering, *E. coli* is not a typical aquatic pathogen. Fundamental differences in ecological niche and environmental adaptation exist between *E. coli* and *V. parahaemolyticus*, making it difficult to directly extrapolate these findings to predict the evolutionary trajectories of pathogens in authentic aquaculture environments. This gap is of particular concern given the increasing prevalence of quinolone resistance in *V. parahaemolyticus* worldwide. Surveillance data indicate a high prevalence of fluoroquinolone non-susceptibility (FQNS) in *V. parahaemolyticus* across Asia and Oceania, with rates ranging from 67% to 97% as of 2019 [25]. Concurrently, although less common, an increasing trend in resistance to third-generation cephalosporins has been noted [26]. This widespread resistance is frequently associated with point mutations in the Quinolone Resistance-Determining Regions (QRDRs) of *gyrA*, *gyrB*, and *parC* genes.

Therefore, this study selected *V. parahaemolyticus* as the research subject. By simulating the antibiotic pressure characteristic of aquaculture environments, the study systematically investigated the phenotypic adaptations and genotypic evolutionary mechanisms of this bacterium under long-term stress from sublethal concentrations of levofloxacin. This research aims to reveal the microevolutionary patterns governing its resistance development, thereby providing a solid theoretical basis for scientifically assessing its ecological and health risks and for formulating precise prevention and control strategies.

## 2. Results

### 2.1. Induction and Heritable Stability Validation of the Quinolone-Resistant Strain VPD14M

**Figure 1:**
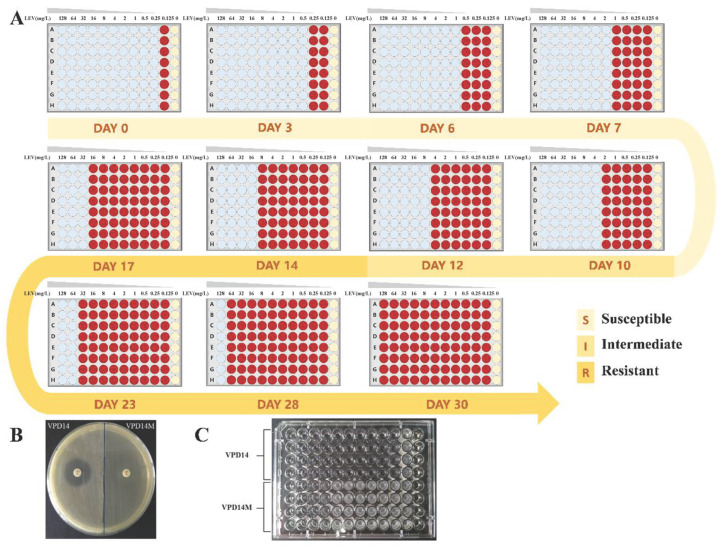
Acquisition and verification of levofloxacin resistance in *V. parahaemolyticus*. (**A**) MIC changes in *V. parahaemolyticus* during levofloxacin induction from day 0 to 30. Blue wells: no growth; Red wells: normal growth; Pale yellow wells: blank control. Verification of levofloxacin resistance in the mutant strain VPD14M using the (**B**) Kirby–Bauer method and (**C**) broth microdilution method.

The antibiotic susceptible *V. parahaemolyticus* VPD14 with a typical quinolone antibiotic levofloxacin MIC of 0.25 mg/L was selected as the initial strain (parental strain), which was inoculated into the MHB culture medium containing different concentrations of levofloxacin for 30 consecutive days, and the change in its resistance profile is shown in Figure 1A. According to CLSI standards, the strain exhibited intermediate resistance (MIC = 4 mg/L) after 10 days of continuous induction. On day 12, the MIC of the parental strain VPD14 reached 8 mg/L, at which point it was classified as resistant and named VPD14M (mutant strain) (Figure 1A). Then the induction was continued for a total of 30 days. As detailed in Figure 1A, the MIC value of the strain increased correspondingly with the induction time. After 30 days of continuous induction under sublethal antibiotic pressure, the results demonstrated that the MIC of the parental strain VPD14 had increased to 256 mg/L. This was a 1024-fold increase compared to the original MIC of 0.25 mg/L for this strain. These results suggested that *V. parahaemolyticus* can evolve resistance to levofloxacin rapidly.

To validate the heritable stability of the acquired resistance, the mutant strain VPD14M was serially passaged for 30 consecutive days in an antibiotic-free medium. Subsequently, its susceptibility was re-evaluated using both the K-B disk diffusion method and the broth microdilution method. The results demonstrated that, in contrast to the parental strain VPD14, the mutant strain VPD14M exhibited no inhibition zone (Figure 1B). Furthermore, the resistance of the mutant strain VPD14M to levofloxacin was markedly higher than that of the parental strain VPD14 (Figure 1C). Moreover, the MIC of the induced *V. parahaemolyticus* met the CLSI-defined resistance breakpoint (MIC ≥ 8 mg/L). These findings confirmed that the mutant strain VPD14M, which possesses stable, heritable levofloxacin resistance, was successfully obtained through this induction process.

### 2.2. Analysis of the Antimicrobial Resistance Profile Changes in the Mutant Strain VPD14M

The broth microdilution method was used to compare the antimicrobial resistance profile of the parental strain VPD14 and the mutant strain VPD14M, in both planktonic and biofilm states (Table 1). The results showed that there were significant differences in the antimicrobial resistance profile of the parental strain VPD14 and the mutant strain VPD14M against common antibiotics. Furthermore, *V. parahaemolyticus* in the biofilm state showed significantly greater resistance to these antibiotics than its planktonic counterparts. In the planktonic state, the antimicrobial resistance profile of the parental strain VPD14 was ampicillin-piperacillin-amikacin, whereas the antimicrobial resistance profile for the mutant strain VPD14M was ampicillin-levofloxacin-ciprofloxacin. In the biofilm state, the antimicrobial resistance profile of the parental strain VPD14 expanded to ampicillin-piperacillin-amikacin-cefazolin, while the antimicrobial resistance profile for the mutant strain VPD14M was ampicillin-piperacillin-levofloxacin-ciprofloxacin. This analysis revealed that the levofloxacin-induced resistant the mutant strain VPD14M also exhibited cross-resistance to ciprofloxacin, another quinolone. However, it lost its resistance to the aminoglycoside amikacin and the penicillin piperacillin. These results revealed distinct differences in the antimicrobial resistance profile between the parental and mutant strains, indicating that the replacement and remodeling of resistance traits occurred during the microevolution of acquired resistance.

### 2.3. Changes in Growth Fitness and Biofilm Formation Capacity

**Figure 2:**
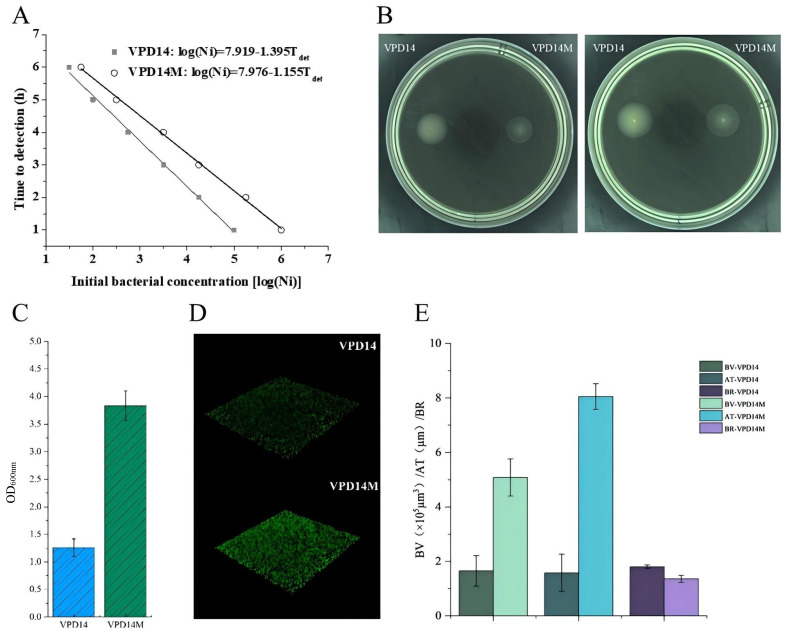
Comparison of biological trait changes between the parental strain VPD14 and the mutant strain VPD14M. (**A**) Growth dynamic parameters of VPD14 and VPD14M; (**B**) motility characteristics of VPD14 and VPD14M; (**C**) biofilm formation capacity of VPD14 and VPD14M; (**D**) confocal laser scanning microscopy (CLSM) images (40× magnification) of biofilms formed by VPD14 and VPD14M; (**E**) biofilm structural parameters of VPD14 and VPD14M.

The growth curves of the parental strain VPD14 and the mutant strain VPD14M were measured at initial concentrations ranging from 10^1^ to 10^5^ CFU/mL using the automated bioscreen growth curve analyzer. Using Origin 8.0 in conjunction with Formula (1), the maximum specific growth rate (*μ_max_*) was calculated, with specific data presented in Figure 2A. The lag phase (*λ*) was then calculated using the obtained parameters and Formula (2). As shown in Table S1, the *μ_max_* of the parental strain VPD14 was 1.40 (log CFU/mL)·h^−1^. After acquiring resistance, its *μ_max_* decreased to 1.20 (log CFU/mL)·h^−1^, representing a significant difference (*p* < 0.05). The *λ* of VPD14 was 1.01 h, which was prolonged to 1.27 h after resistance acquisition (*p* < 0.05). This indicated that the acquisition of resistance resulted in reduced growth fitness. The motility of the parental strain VPD14 and the mutant strain VPD14M were also assessed on 0.3% LB semi-solid agar (Figure 2B). This assay also revealed that the growth and motility of the strains were reduced after acquiring resistance. Both the parental strain VPD14 and the mutant strain VPD14M exhibited motility on the semi-solid medium. However, the parental strain VPD14 formed a larger, opaque swarm, whereas the mutant strain VPD14M produced a smaller, translucent swarm. This demonstrated that the growth and motility of the parental strain VPD14 were markedly superior to those of the mutant strain VPD14M. This finding is consistent with the bioscreen results, both methods indicated that the parental strain VPD14 possesses greater growth fitness than the mutant strain VPD14M.

According to the biofilm formation classification criteria, the mutant strain VPD14M, with an optical density at 600 nm (OD_600nm_) greater than 4ODc, was classified as a strong biofilm producer. The parental strain VPD14, with an OD_600nm_ between 2ODc and 4ODc, was classified as a moderate biofilm producer. Under identical culture conditions, the amount of biofilm formed by the mutant strain VPD14M was significantly greater than that formed by the parental strain VPD14 (Figure 2C). VPD14M exhibited a dense, adherent biofilm structure, whereas VPD14 formed a relatively sparse, monolayer biofilm (Figure 2D). Analysis of the three-dimensional structural parameters of biofilm using ISA software (version 2.0) (Figure 2E) revealed that the mutant strain VPD14M showed a significant increase in biovolume (BV) and average thickness (AT), but a decrease in biofilm-roughness (BR), compared to the parental strain VPD14 (Table 2). This result indicated that after *V. parahaemolyticus* acquired resistance, the interstitial voids within its biofilm were reduced, and the surface became smoother.

### 2.4. Genetic Mechanisms of Quinolone Resistance

**Figure 3:**
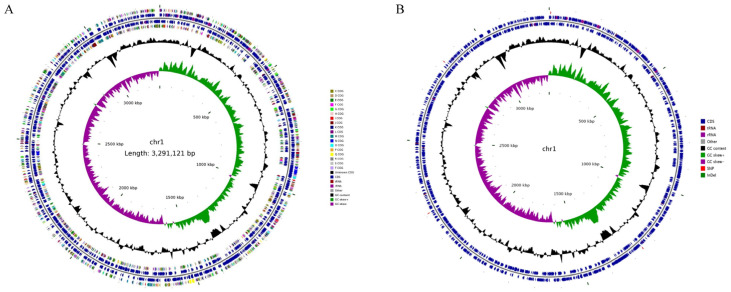
Genomic analysis of the parental strain VPD14 and the mutant strain VPD14M. The figure shows a circular representation of the wild-type chr1 of the parental strain VPD14 genome (**A**) and chr1 with non-synonymous SNPs and InDels (**B**). From the inside out, the first circle represents the scale; the second circle represents the GC skew; the third circle represents the GC content; the fourth and seventh circles represent the Clusters of Orthologous Groups (COGs) to which each coding sequence (CDS) belongs; the fifth and sixth circles represent the corresponding positions of CDS, tRNA, and rRNA on the genome.

To identify Single-Nucleotide Polymorphisms (SNPs) potentially responsible for the levofloxacin resistance mechanism, the complete genome of the parental strain VPD14 (Table S2) was used as the reference. The genomic differences between the parental strain VPD14 and the mutant strain VPD14M were analysed using comparative genomics. The genome of the parental strain VPD14 consisted of two circular chromosomes (chr1 and chr2), with lengths of 3,291,121 bp and 1,877,519 bp, respectively (Figure 3A). The GC contents for these two chromosomes were 45.38% and 45.36%, respectively (Table S3). The genome length of the mutant strain VPD14M was 5,089,287 bp, with a GC content of 45.29% (Table S4 and Figure 3B). Further analysis involved screening and annotation to identify Insertion–Deletion (InDel) sites, with the results shown in Table S5. Regarding Copy Number Variations (CNVs), no sequence duplications were found, although single sequence deletions were present (Table S6). Notably, the chromosomal structure of the mutant strain VPD14M exhibited only deletion variations; no insertions, inversions, intra-chromosomal translocations, or inter-chromosomal translocations were detected (Table S7).

### 2.5. Mutations in gyrA, gyrB, and parC Confer Quinolone Resistance to Mutant Strain VPD14M

**Figure 4:**
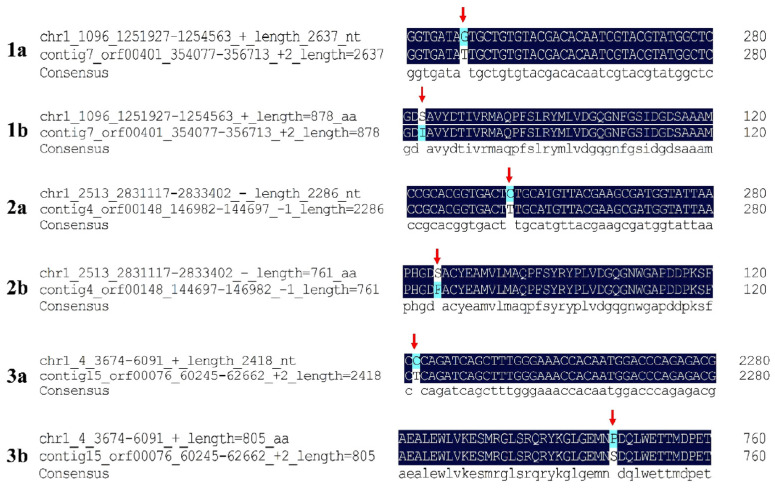
Nucleotide substitutions and corresponding amino acid changes in the resistance genes *gyrA*, *gyrB*, and *parC*. Panels (**1a**,**2a**,**3a**) show the nucleotide sequences, and panels (**1b**,**2b**,**3b**) show the amino acid sequences.

A statistical analysis of antibiotic resistance genes (ARGs) was performed to identify the genetic changes specific to the parental strain VPD14 and the mutant strain VPD14M (Table S8). The results showed that the parental strain VPD14 carried a total of 50 ARGs, whereas the mutant strain VPD14M carried only 48 ARGs. Among these 48 resistance genes, only three genes showed sequence similarities of less than 100% (Table S9, Figure 4). Specifically, the *gyrA* gene, which encodes the DNA gyrase subunit A, exhibited a mutation from base G to T at position 248, corresponding to a non-synonymous Single-Nucleotide Polymorphism (nSNP) at position 1,252,174 (Figure 4(1a)). This mutation resulted in the substitution of serine (Ser) with isoleucine (Ile) at the 83rd amino acid position of GyrA (Figure 4(1b)). Similarly, the *parC* gene, encoding the DNA topoisomerase IV subunit A, showed a mutation from base C to T at position 254, corresponding to an nSNP at position 2,833,149 (Figure 4(2a)). This mutation led to the substitution of serine (Ser) with phenylalanine (Phe) at the 85th amino acid position of ParC (Figure 4(2b)). Furthermore, the *gyrB* gene, encoding the DNA gyrase subunit B, had a mutation from base C to T at position 2242, corresponding to an nSNP at position 5915 (Figure 4(3a)). This mutation caused the substitution of proline (Pro) with serine (Ser) at the 748th amino acid position of GyrB (Figure 4(3b)). It was observed that the mutant strain VPD14M carried quinolone resistance genes, including *gyrA*, *gyrB*, *parC*, *parE*, *qnrVC1*, and *mdtK*. However, no changes were detected in the *parE*, *qnrVC1*, or *mdtK* genes. Therefore, under the stimulation of sublethal antibiotic concentrations, mutations in the quinolone resistance genes *gyrA*, *gyrB*, and *parC* conferred quinolone resistance to the mutant strain VPD14M.

## 3. Discussion

The persistent residues of antibiotics in aquaculture environments, particularly the environmental selection pressure posed by sublethal concentrations of quinolones, are continuously driving the evolution of resistance in aquatic pathogens [4,5,6,11]. Although surface water quinolone levels are typically in the ng/L–μg/L range, quinolones accumulate in sediments up to 4 mg/kg due to high adsorption [12,27,28]. In this study, the initial sublethal concentration of 0.125 mg/L (1/2 MIC of the parental strain) was selected to simulate bioavailable antibiotic levels in sediment pore water, a realistic scenario given that quinolones can accumulate in sediments at concentrations up to 4 mg/kg. This concentration, approximating 1/30 of the peak sediment residue, models the chronic, low-level selective pressure exerted on *V. parahaemolyticus* in benthic aquaculture environments. The results confirmed that exposure to environmentally relevant concentrations of antibiotics can induce stable, heritable, high-level quinolone resistance in *V. parahaemolyticus* within a very short timeframe (30 days), resulting in an MIC increase of up to 1024-fold. This finding provides direct experimental evidence for understanding the rapid emergence of resistance in aquaculture environments (Figure 1).

Compared to studies conducted using model organisms such as *E. coli* [23], this study observed more complex phenotypic adaptive trade-offs in an authentic aquatic pathogen. The acquisition of quinolone resistance in *V. parahaemolyticus* was concurrent with a significant fitness cost, manifested as a notable decrease in the maximum specific growth rate (*μ_max_*) and motility (Figure 2A). While the reduction in these absolute growth kinetic parameters suggests that the mutant strain may possess a lower relative fitness compared to its parental counterpart in antibiotic-free environments, this growth disadvantage was strategically offset by a significant compensatory adaptation, a remarkable enhancement of biofilm formation capacity. The observed fitness trade-offs likely originate from global transcriptional reprogramming and a re-allocation of cellular resources [29]. The fixed mutations in *gyrA* and *parC* are predicted to alter DNA supercoiling dynamics, a master regulator of gene expression. This can dysregulate large regulons governing core metabolism and flagellar biosynthesis, effectively imposing a physiological constraint on growth and motility. In response, the cell appears to activate stress-adaptive pathways, diverting energy toward biofilm matrix production as a compensatory survival strategy [1,3,30]. To compensate for this fitness cost under sustained antibiotic stress, *V. parahaemolyticus* appears to activate stress-response pathways, such as the SOS response and RpoS-mediated networks, which facilitate a transition from a motile, planktonic lifestyle to a sessile, biofilm-protected state [31]. This shift is often governed by the c-di-GMP signaling pathway, which acts as a molecular switch prioritizing the synthesis of extracellular polymeric substances (EPS) over flagellar synthesis [2,4,32].

Intriguingly, this regulatory ‘switch’ not only drives structural remodeling but also governs the observed remodeling of the resistance phenotype, where the acquisition of quinolone resistance occurred alongside the collateral loss of resistance to amikacin and piperacillin [33]. Such modifications are likely directed by the differential expression of resistance-related genes or stress-response pathways triggered by chronic antibiotic exposure [34,35]. Specifically, ‘genomic pruning’, whereby non-essential resistance determinants are removed, such as transcriptional repression of efflux pumps or loss of mobile genetic elements carrying aminoglycoside-modifying enzymes, may alleviate the metabolic burden of maintaining costly QRDR mutations [36]. Furthermore, the quinolone-induced SOS response may create a metabolic bottleneck that prioritises DNA repair and matrix synthesis over cell-wall modification pathways linked to piperacillin resistance [30,31,37]. This compensatory strategy that ensures survival in contaminated aquaculture environments despite the inherent growth disadvantage.

The structural transition toward a thicker, more compact biofilm in VPD14M, characterized by increased biovolume (BV) and reduced roughness (BR), suggests a fundamental reprogramming of biofilm-regulatory networks under chronic quinolone stress (Table 2) [38,39]. The enhanced biofilm architecture is likely mediated by the upregulated production of key matrix components, notably extracellular polysaccharides (EPS) and DNA (eDNA). This process is often orchestrated by master regulatory systems, such as the vpS cluster and the general stress response sigma factor RpoS, which are induced under antibiotic stress [1,3,31,40]. Furthermore, the smoother, thicker biofilm morphology suggests a transition to a more communal lifestyle, facilitated by heightened levels of the second messenger c-di-GMP. This molecule typically represses genes for motility while activating those for expolymeric substance synthesis, leading to the observed structural consolidation [2,4,32]. Furthermore, the enrichment of eDNA may be linked to the differential expression of genes involved in controlled cellular autolysis or the SOS response [31], which is triggered by quinolone-induced DNA damage and has been shown to enhance biofilm maturation [5,37]. The distinct discrepancy in resistance profiles between planktonic and biofilm states, notably the increased resistance to piperacillin and cefazolin in the latter, can be attributed to the unique microenvironment of the matrix (Table 1). Mechanistically, the overproduced extracellular polymeric substances (EPS) in VPD14M act as a physical and chemical shield, sequestering antibiotic molecules and limiting their penetration [38,39]. Beyond this physical barrier, the biofilm architecture fosters metabolic heterogeneity, where oxygen and nutrient gradients induce a state of reduced metabolic activity in basal cells, rendering them inherently more tolerant to antibiotics that target active cellular processes [32]. Collectively, such architectural and physiological modifications represent a critical compensatory strategy that ensures the survival and persistence of *V. parahaemolyticus* in contaminated aquaculture environments, effectively mitigating the inherent growth disadvantages associated with high-level resistance.

At the molecular level, this study precisely elucidated the key genetic events driving this microevolutionary process [23]. Unlike the evolutionary paths largely inferred from model organisms, this study demonstrated that in *V. parahaemolyticus*, high-level quinolone resistance is primarily determined by the combination of specific non-synonymous mutations in three key genes, *gyrA*, *parC*, and *gyrB* (Figure 4). The stepwise, incremental rise in MIC observed over the 30-day induction period, from 0.25 mg/L to 256 mg/L, implies a sequential accumulation of genetic alterations, rather than a single mutational event. This pattern contrasts with findings in *E. coli*, where sublethal fluoroquinolone exposure sometimes leads to resistance primarily through mutations in *gyrA* [41,42]. The involvement of three QRDR genes (*gyrA*, *parC*, and *gyrB*) in our mutant suggests that *V. parahaemolyticus* may require a more complex genetic adaptation for high-level resistance, possibly involving compensatory interactions between targets [23].

Our genomic screening confirmed target-site remodeling as the primary stable genetic basis, as no fixed alterations were found in efflux pump or porin regulators. However, the remarkable speed of this evolution raises the question of whether sublethal stress induced a hypermutator phenotype. Although not assessed here via fluctuation assays, determining whether stress-enhanced mutation rates accelerated this trajectory is a promising direction for future research. Additionally, the genomic deletion variants identified in VPD14M likely represent an auxiliary ‘streamlining’ strategy to optimize fitness, which deserves further functional characterization through site-directed mutagenesis. Finally, future studies should employ competition assays under subinhibitory fluoroquinolone concentrations to quantitatively assess the relative fitness and competitive success of mutant strains compared with their parental counterparts in diverse ecological niches.

## 4. Materials and Methods

### 4.1. Strains, Antibiotics, and Antimicrobial Susceptibility Screening

The antibiotic susceptible *V. parahaemolyticus* VPD14 was isolated from seafood [24]. *E. coli* ATCC 25922, as a quality control strain, was purchased from the China Center of Industrial Culture Collection (CICC, Beijing, China). Levofloxacin was purchased from (Shanghai Sigma Aldrich Trading Co., Ltd., Shanghai, China). VPD14 was retrieved from −80 °C with Thiosulfate-Citrate-Bile Salts-Sucrose agar (TCBS, Beijing Land Bridge Technology Co., Ltd., Beijing, China) at 37 °C for 24 h. And then a single colony was of VPD14 was inoculated into 10 mL of Tryptic Soy Broth (TSB, 3% NaCl; Beijing Land Bridge Technology Co., Ltd., Beijing, China) at 37 °C for 16. The bacterial suspension was adjusted to 1.5 × 10^8^ CFU/mL, was then inoculated into 96-well plates containing serial two-fold dilutions of levofloxacin (final concentrations of 128, 64, 32, 16, 8, 4, 2, 1, 0.5, and 0.25 µg/mL) at 37 °C for 16 h. The resistance profile of VPD14 was determined by the Clinical and Laboratory Standards Institute (CLSI, 2018).

### 4.2. Acquisition of Quinolone Resistance in VPD14

The MIC of VPD14 was measured using the microdilution method in Mueller–Hinton broth (Beijing Land Bridge Technology Co., Ltd., Beijing, China), as recommended by the Clinical and Laboratory Standards Institute (CLSI, 2018). The bacterial suspension was adjusted to 1 × 10^6^ CFU/mL with 0.85% NaCl (Sangon Biotech (Shanghai) Co., Ltd., Shanghai, China). This bacterial suspension was then inoculated into 96-well plates containing various concentrations of levofloxacin and incubated at 37 °C for 16 h. Notably, to rapidly obtain a genetically stable resistant mutant (VPD14M), the parental strain VPD14 was continuously cultured at a sublethal concentration (1/2 MIC) for 12–30 days, until its MIC against the antibiotic reached the resistance breakpoint defined by CLSI. The mutant was streaked onto Mueller–Hinton agar (MHA, Oxoid, Basingstoke, UK) plates containing 8 mg/L levofloxacin and incubated at 37 °C for 16 h. Subsequently, colonies were inoculated into 9 mL TSB tubes (3% NaCl). The tubes were incubated at 37 °C for 16 about 200 rpm. After incubation, 10 µL of the bacterial suspension was transferred into 9 mL of fresh TSB (3% NaCl) and incubated under the same conditions (37 °C, 200 rpm) for 16 h. This procedure was repeated for 5 consecutive days until the stability of the acquired resistance in the mutant.

### 4.3. K-B Disk Diffusion

*E. coli* ATCC 25922 was used as the quality control strain. The K-B disk diffusion was employed to test the levofloxacin susceptibility of the resistant mutant VPD14M. A bacterial suspension was adjusted to 1.5 × 10^8^ CFU/mL. A cotton swab was dipped into the suspension and used to evenly inoculate the entire surface of Mueller–Hinton agar (Beijing Land Bridge Technology Co., Ltd., Beijing, China). After the inoculum on the medium surface was allowed to dry completely, the antibiotic disks were applied. The plates were then placed in a 37 °C incubator for 16 h, and the results were observed and recorded. In accordance with CLSI standards, the diameters of the inhibition zones were measured, and the *V. parahaemolyticus* was classified as resistant, intermediate, or susceptible.

### 4.4. Determination of Growth Kinetics

The growth kinetic parameters of VPD14 and VPD14M were determined using a bioscreen assay. The specific method was as follows: 180 μL of TSB medium (3% NaCl, pH = 8) was added to six wells of a bioscreen-100 microplate. An initial inoculum (20 μL, 1 × 10^7^ CFU/mL) was added to the first well. A serial dilution was then performed by transferring 20 μL from the first well to the second well, and this process was repeated to obtain bacterial suspensions with initial concentrations ranging from 10^6^ to 10^1^ CFU/mL. TSB medium without inoculum served as the blank control. The 100-well microplate was placed into the bioscreen instrument, which was set to read the OD_600_ value every 15 min at an incubation temperature of 37 °C. Growth curves were measured for VPD14 and VPD14M. Each experiment was performed in triplicate.

### 4.5. Calculation of Growth Kinetic Parameters

The formulas used to calculate the growth kinetics of *V. parahaemolyticus* from the bioscreen data are as follows:

Formula for maximum specific growth rate:

(1) $$log(N_{ini})=K-\mu_{max}\times T_{det}$$

Formula for the lag phase:

(2) $$\lambda =[K-log(N_{det})]/\mu_{max}$$

where *T_det_* is the time (h) required for the bacterial concentration to reach the detectable level of the bioscreen (10^6.5^ CFU/mL); *μ_max_* is the maximum specific growth rate [(log CFU/mL)·h^−1^]; *N_ini_* is the initial bacterial concentration (CFU/mL); *N_det_* is the initial bacterial concentration (CFU/mL) corresponding to *T_det_*, K is a parameter obtained from the formula fitting.

### 4.6. Motility Assay

A TCBS plate with well-grown, isolated colonies was selected. Single colonies of VPD14 and VPD14M were picked with a sterile toothpick and inoculated into the centre of 0.3% LB semi-solid agar plates in parallel at 37 °C. After 4 h, the differences in motility between VPD14 and VPD14M were observed and photographed using a colony counter.

### 4.7. Biofilm Culture

The bacterial suspension was diluted with 0.85% physiological saline, and the OD was adjusted to OD_600_ = 0.6. In a 24-well plate, 990 μL of fresh TSB (3% NaCl) medium was added to each well, followed by 10 μL of the diluted bacterial suspension. The final column, containing uninoculated fresh TSB, served as a blank control. Five parallel wells (replicates) were prepared for each strain. The 24-well plate was sealed with plastic wrap and placed in a 25 °C incubator for static culture for 48 h. Following incubation, the quantity of biofilm formation was measured, and the biofilm structure was observed.

### 4.8. Crystal Violet Staining

After 48 h of incubation, the 24-well plates were removed, and the planktonic cells in the supernatant were discarded. Each well was washed three times with 0.1 mol/L PBS buffer (Shanghai Sangon Bioengineering Co., Ltd., Shanghai, China). Then the adherent biofilms were stained with 0.1% crystal violet (Sigma Aldrich (Shanghai) Trading Co., Ltd., Shanghai, China) for 30 min. After 30 min, the crystal violet solution was slowly aspirated. To remove excess dye, the wells were gently washed three times with PBS buffer and then allowed to air dry for 30 min. Following drying, 1 mL of 95% ethanol (AR, Shanghai sinopharm Chemical Reagents Co., Ltd., Shanghai, China) was added to each well to dissolve the bound dye for 15 min. Subsequently, 200 μL of the dissolved solution was transferred to a 96-well plate, and the absorbance value at 600 nm (OD_600nm_) was measured using a microplate reader. Uninoculated fresh TSB served as the blank control (ODc). The biofilm-forming capacity was classified into four levels based on the following criteria: no biofilm formation (OD_600nm_ < ODc); weak biofilm formation (ODc < OD_600nm_ ≤ 2ODc); moderate biofilm formation (2ODc < OD_600nm_ ≤ 4ODc); and strong biofilm formation (OD_600nm_ > 4ODc).

### 4.9. CLSM Analysis of Biofilm Structure

Sterile, clean glass coverslips were placed into a 24-well plate for biofilm cultivation. After the biofilms reached maturity, the culture medium was aspirated from the wells, and the coverslips were gently washed three times with 0.1 mol/L PBS. Following washing, 2 mL of 4% (*v*/*v*) glutaraldehyde (Shanghai Sangon Bioengineering Co., Ltd., Shanghai, China) was added to each well for fixation at a low temperature for 30 min. Excess glutaraldehyde was then removed by washing with 0.1 mol/L PBS buffer. Next, the biofilms were stained with SYBR Green I dye (Beijing Solebo Technology Co., Ltd., Beijing, China) for 30 min at room temperature in the dark. After staining, the coverslips were gently washed with PBS and allowed to dry. Finally, the biofilm structure was observed using a confocal laser scanning microscope (CLSM, Carl Zeiss, Jena, Germany). Biofilm structural parameters were analyzed using Zeiss LSM Image Browser software (v. 4.0.121, Carl Zeiss). Results were expressed as mean ± standard deviation.

### 4.10. Statistical Analysis of Biofilm Structural Parameters

Graphing was performed using Origin Pro 9.1, and SPSS 20.0 software was used for comparative analysis. Differences between samples were considered statistically significant (*p* < 0.05). Biovolume (BV), average thickness (AT), and biofilm roughness (BR) were generated using the Image Structure Analyzer-2 (ISA-2), provided by Professor Haluk Beyenal of Montana State University, USA. All experiments pertaining to antimicrobial susceptibility testing, growth kinetics, motility assays, and biofilm quantification were performed with a minimum of three independent biological replicates. Data are presented as the mean ± standard deviation.

### 4.11. DNA Extraction

Total genomic DNA was extracted from VPD14 and VPD14M using a bacterial genomic DNA kit (Tiangen Biochemical Technology Co., Ltd., Beijing, China). The concentration and quality of the extracted DNA samples were assessed using a BioTek multifunction microplate reader (Biotek Instruments, Winooski, VT, USA) and verified by 0.9% agarose gel electrophoresis.

### 4.12. Sequencing

Insert-fragment libraries for VPD14 and VPD14M were constructed using a Whole Genome Shotgun (WGS) approach. These libraries were sequenced using both the Illumina MiSeq platform (a Next-Generation Sequencing, NGS, technology) for paired-end (PE) sequencing and the PacBio platform (a third-generation single-molecule sequencing technology). Detailed information on the library construction for the sequencing of VPD14 and VPD14M is provided in Table S2.

### 4.13. Genome Sequence Assembly and Analysis

The next-generation sequencing (NGS) data for VPD14 and VPD14M were assembled using A5-miseq (version 20160825) and A5-miseq (v20150522) software, respectively. Additionally, the Kmer-corrected data were assembled using SPAdes genome assembler (v3.11.1) to obtain contig and scaffold sequences. For the third-generation single-molecule sequencing data of strain VPD14, the raw data obtained from PacBio sequencing were assembled using HGAP4 software (version 4.0) to generate scaffold sequences. The contigs derived from both the NGS and third-generation assemblies were then subjected to a collinearity analysis using MUMmer (v3) software. This step confirmed the assembly results, determined the positional relationships between contigs, and facilitated gap filling. The resulting assembly was further corrected using Pilon (version 1.22) to finalize the complete sequence of VPD14. Finally, the quality of the assembly was evaluated for the complete genome sequence of VPD14 and for the contig and scaffold files of VPD14M.

### 4.14. Comparative Genomics Analysis of Parental Strain VPD14 and Mutant Strain VPD14M

The bwa (0.7.12-r1039) aln program was used to compare the filtered high-quality data to the reference genome, with the comparison parameters following the default parameters of bwaaln. The sai files obtained from the comparison were transformed into sam files using bwa sampe and further transformed into bam file format using the samtools (0.1.19-44428cd) toolkit. Sorting was performed using picard 1.107 (http://www.psc.edu/index.php/user-resources/software/picard, accessed on 8 January 2018) software to ensure that all pairs of ends read the same information. All near-InDel reads were reconsidered using GATK (version 4.0) (https://www.broadinstitute.org/gatk/download/, accessed on 8 January 2018) software to improve prediction accuracy. Similarly, the InDel loci for each sample were obtained using GATK and subjected to SNP detection. All detected SNPs were then labeled using Annovar software (version 2018Apr16). Genome-wide CNVs were detected using CNVnator V0.2.7, possible CNV regions were obtained, and genes in these CNV regions were extracted and annotated. Possible chromosomal structural variants in the genome were detected using Breakdancer 1.3.7. Non-synonymous mutation information (including SNPs and InDel) was extracted and mapped using CGView (http://cgview.ca/, accessed on 8 January 2018).

### 4.15. Antibiotic Resistance Analysis of Parental Strain VPD14 and Mutant Strain VPD14M

The database used in this study is The Comprehensive Antibiotic Resistance Database (CARD), and antibiotic resistance analysis can be used to obtain genomic sequences of the antibiotic resistance genes. BLAST software (version 2.6.0+) was used to predict the genes related to antibiotic resistance present in the genome, with BLAST comparison set parameters E-value of 1 × 10^−6^, amino acid sequence identity of 45% or more, and the ratio of the length of the sequence comparison to its sequence length not less than 70%.

## 5. Conclusions

In conclusion, this study confirmed that sublethal concentrations of quinolone residues in aquaculture systems are sufficient to drive the rapid microevolution of resistance in *V. parahaemolyticus*. This process involves precise genetic mutations and complex phenotypic adaptive remodeling. Given that *V. parahaemolyticus* can directly threaten human health via contaminated aquatic products, the rapid evolution of its resistance exacerbates the public health risks posed by AMR [23,24]. Based on our elucidated microevolutionary pathway, we propose a tiered molecular surveillance strategy for proactive resistance monitoring. We recommend that the GyrA-Ser83Ile mutation serve as a primary ‘sentinel’ marker for incipient resistance. Subsequently, the concurrent detection of ParC-Ser85Phe and GyrB-Pro748Ser mutations should flag high-risk isolates that have evolved stable, high-level resistance, warranting heightened vigilance. Therefore, curbing the evolution of resistance in *V. parahaemolyticus* necessitates a synergistic governance strategy based on the “One Health” concept. This includes strict control over antibiotic use in aquaculture and enhanced cross-sectoral surveillance of resistant pathogens across the environment–animal–food chain. Such measures are essential to fundamentally interrupt the transmission chain of resistance between the environment, animals, and humans, thereby safeguarding both ecological integrity and public health.

## Figures and Tables

**Table 1:** The antimicrobial resistance profile of inconsistent forms of *V. parahaemolyticus*.

State	Strain	Antimicrobial Resistance Profile
Planktonic	VPD14	AMP, PRL, AK
	VPD14M	AMP, LEV, CIP
Biofilm	VPD14	AMP, PRL, AK, KZ
	VPD14M	AMP, PRL, LEV, CIP

**Table 2:** The three-dimensional structural parameters of biofilm in *V. parahaemolyticus*.

Strain	OD_600nm_	BV (×10^5^ µm^3^)	AT (µm)	BR
VPD14M	3.834 ± 0.27	5.08 ± 0.68	8.05 ± 0.47	1.36 ± 0.13
VPD14	1.258 ± 0.16	1.65 ± 0.56	1.58 ± 0.68	1.80 ± 0.07

## Data Availability

The SRA records presented in the study are openly available in [National Center for Biotechnology Information] at (https://www.ncbi.nlm.nih.gov/sra/PRJNA906012, access on 8 January 2018).

## Supplementary Materials

The following supporting information can be downloaded at https://www.mdpi.com/article/10.3390/ijms27031416/s1.
