# Supplementary material for: Sublethal Antibiotic Exposure Induces Microevolution of Quinolone Resistance in Pathogenic Vibrio parahaemolyticus
Source: Int J Mol Sci. 2026 Jan 30;27(3):1416. doi: 10.3390/ijms27031416 (PMC12898516; doi:10.3390/ijms27031416)
Supplement: Supplementary file 1 [file ijms-27-01416-s001.zip › ijms-4048520-supplementary.pdf]

Table S1. The parameters of growth dynamics between *Vibrio parahaemolyticus* the sensitive VPD14 and the resistant mutants VPD14M.

Growth parameters	VPD14	VPD14M
The maximum growth rate [μ_{max}, (log CFU/g) • h^{-1}]	1.40±0.04	1.20±0.02
The average lag phases (λ, h)	1.01±0.10	1.27±0.08

Note: Experimental data are expressed as mean ± standard deviation; a different superscript lowercase letter in the peer group indicates a significant difference between the two ($p<0.05$), and the same superscript lowercase letter in the peer group indicates a non-significant difference ($p>0.05$).

Table S2. Overview of sequencing strategy.

Sample	Lib.Name	Lib.Insert Size	Sequencing platform	Sequencing Mode
VPD14	S20K	20kb	PacBio	Standard
VPD14	PE400	400bp	Illumina Miseq	Paired-end, 2X251bp
VPD14M	PE400	400bp	Illumina Miseq	Paired-end, 2X251bp

Table S3. The data statistics of genome assembly of VPD14.

Sample	Seq ID	Seq Length(bp)	GC Content (%)	Seq Type
VPD14	Chr1	3,291,121	45.38	circular
VPD14	Chr2	1,877,519	45.36	circular

Table S4. The data statistics of genome assembly of VPD14M.

Sample	Property	Contig	Scaffold
	Shortest(bp)	1,035	1,035
	Longest(bp)	844,892	844,892
	Total sequence number	32	31
	N20(bp)	580,697	614,387
	N50(bp)	466,045	446,045
VPD14M	N90(bp)	101,089	123,239
	N number	0	366
	N rate	0	0.0001
	Total sequence length	5,089,287	5,089,287
	GC content	45.29	45.29
	Sequence greater than 1kb	32	31

Table S5. The annotation results of InDel.

Sample	VPD14M
Count	5
Upstream	5
Downstream	0
Upstream; Downstream	0
Exonic	0
ncRNA-exonic	0
UTR5	0
Frameshift deletion	0
Frameshift insertion	0
Nonframeshift deletion	0
Nonframeshift insertion	0
Stopgain	0
Stoploss	0
unknown	0

Table S6. The number of CNV.

Sample	Duplication Num.	Deletion Num.
VPD14M	0	1

Table S7. The number of structural variation of chromosomes.

Sample	DEL	INS	INV	ITX	CTX
VPD14M	1	0	0	0	0

Note: DEL: Deletion; INS: Insertion; INV: Inversion; ITX: Intrachromosomal translocation; CTX: Interchromosomal translocation

Table S8. The summary of resistance genes in *V. parahaemolyticus*.

Sample	Property	Number of genes	Percentage (%)
VPD14			
chr1	Antibiotic resistance	23	0.778
	Antibiotic target	20	0.677
	Antibiotic biosynthesis	2	0.068
	Total genes	38	1.286
chr2	Antibiotic resistance	9	0.532
	Antibiotic target	3	0.177
	Antibiotic biosynthesis	0	0
	Total genes	12	0.71
VPD14M	Antibiotic resistance	30	0.644
	Antibiotic target	23	0.493
	Antibiotic biosynthesis	2	0.043
	Total genes	48	1.030

Table S9. The summary of resistance genes in *V. parahaemolyticus*.

Sample ID		Product	Identity
VPD14	VPD14M		
chr1_126	-	His-Xaa-Ser repeat protein HxsA	0.00%
chr1_276	-	methyl-accepting chemotaxis protein	0.00%
chr2_161	contig5_orf00052	beta-lactamase	86.62%
chr1_1096	contig7_orf00401	DNA gyrase subunit A	99.96%
chr1_2513	contig4_orf00148	DNA topoisomerase IV subunit A	99.96%
chr1_4	contig15_orf00076	DNA gyrase subunit B	99.96%
chr2_1225	contig3_orf00259	multidrug efflux system protein	100%
chr2_1394	contig3_orf00473	chloramphenicol acetyltransferase	100%
chr2_1487	contig3_orf00590	QnrVC1. Confers resistance to fluoroquinolones	100%
chr2_136	contig5_orf00020	Catalase-peroxidase-peroxynitritase T KatG	100%
chr2_155	contig5_orf00044	Resistance-Nodulation-cell Division (RND) multi-drug efflux transporter MexF	100%
chr2_1616	contig9_orf00103	MacB. ABC transporter	100%
chr2_25	contig9_orf00244	redox-sensitive transcriptional activator of soxS	100%
chr2_435	contig5_orf00419	Catalase-peroxidase-peroxynitritase T KatG	100%
chr2_552	contig5_orf00559	D-alanine--D-alanine ligase	100%
chr2_663	contig6_orf00380	putative alanine racemase	100%
chr2_20	contig9_orf00235	putative translation elongation factor G	100%
chr1_2812	contig4_orf00491	GTP-binding protein TypA	100%
chr1_1846	contig1_orf00494	global DNA-binding transcriptional dual regulator H-NS	100%
chr1_2925	contig15_orf00034	acetolactate synthase 2 catalytic subunit	100%
chr1_2610	contig4_orf00261	50S ribosomal protein L21	100%
chr1_1886	contig1_orf00544	Inner membrane transporter; multidrug efflux pump	100%
chr1_729	contig8_orf00061	1-deoxy-D-xylulose 5-phosphate reductoisomerase	100%
chr1_475	contig8_orf00370	signal peptidase I	100%
chr1_2587	contig4_orf00235	acetolactate synthase 3 catalytic subunit	100%
chr1_2698	contig4_orf00365	UDP-glucose 6-dehydrogenase	100%
chr1_195	contig12_orf00038	sensory histidine kinase in two-component regulatory system with CpxR	100%
chr1_860	contig7_orf00098	hypothetical protein	100%
chr1_2519	contig4_orf00155	TolC. Multi-drug efflux pump	100%
chr1_927	contig7_orf00177	electron transport complex subunit E	100%
chr1_434	contig8_orf00414	alanine racemase family protein	100%
chr1_583	contig8_orf00240	7,8-dihydropteroate synthase	100%
chr1_2282	contig11_orf00141	tet34 curated	100%
chr1_1845	contig1_orf00493	tet35 protein	100%
chr1_2782	contig4_orf00458	transcription elongation factor GreB	100%
chr1_255	contig12_orf00121	DNA-binding transcriptional dual regulator	100%
chr1_579	contig8_orf00244	transcription elongation factor GreA	100%
chr1_616	contig8_orf00196	translation elongation factor G-like protein	100%
chr1_2605	contig4_orf00256	dihydrofolate reductase	100%
chr1_205	contig12_orf00053	alanine racemase	100%
chr1_273	contig12_orf00144	30S ribosomal protein S12	100%
chr1_196	contig12_orf00040	response regulator in two-component regulatory system with CpxA	100%

chr1_1514	contig1_orf00092	multidrug efflux system transporter	100%
chr1_2514	contig4_orf00149	DNA topoisomerase IV subunit B	100%
chr1_1887	contig1_orf00545	putative membrane fusion protein	100%
chr1_312	contig17_orf00019	alanine racemase biosynthetic	100%
chr1_134	contig18_orf00009	DNA-directed RNA polymerase beta subunit	100%
chr1_275	contig12_orf00146	translation elongation factor G	100%
chr1_133	contig18_orf00008	DNA-directed RNA polymerase beta subunit	100%
chr1_2891	contig4_orf00594	Resistance-Nodulation-cell Division (RND) efflux transporter	100%
